# Maternal, placental and cord blood cytokines and the risk of adverse birth outcomes among pregnant women infected with *Schistosoma japonicum* in the Philippines

**DOI:** 10.1371/journal.pntd.0007371

**Published:** 2019-06-12

**Authors:** Ajibola I. Abioye, Emily A. McDonald, Sangshin Park, Ayush Joshi, Jonathan D. Kurtis, Hannah Wu, Sunthorn Pond-Tor, Surendra Sharma, Jan Ernerudh, Palmera Baltazar, Luz P. Acosta, Remigio M. Olveda, Veronica Tallo, Jennifer F. Friedman

**Affiliations:** 1 Center for International Health Research, Rhode Island Hospital, The Warren Alpert Medical School of Brown University, Providence, RI, United States of America; 2 Department of Pediatrics, The Warren Alpert Medical School of Brown University, Providence, RI, United States of America; 3 Graduate School of Urban Public Health, University of Seoul, Seoul, Republic of Korea; 4 Department of Pathology and Laboratory Medicine, The Warren Alpert Medical School of Brown University, Providence, RI, United States of America; 5 Department of Pediatrics, Women and Infants Hospital of Rhode Island, Providence, RI, United States of America; 6 Departments of Clinical Immunology and Transfusion Medicine, Linkoping University, Linkoping, Sweden; 7 Departments of Clinical and Experimental Medicine, Linkoping University, Linkoping, Sweden; 8 Remedios Trinidad Romualdez Hospital, Tacloban City, Leyte, The Philippines; 9 Research Institute for Tropical Medicine, Manila, Philippines; University of Cambridge, UNITED KINGDOM

## Abstract

**Background:**

The objectives of this study were to 1) evaluate the influence of treatment with praziquantel on the inflammatory milieu in maternal, placental, and cord blood, 2) assess the extent to which proinflammatory signatures in placental and cord blood impacts birth outcomes, and 3) evaluate the impact of other helminths on the inflammatory micro environment.

**Methods/Findings:**

This was a secondary analysis of samples from 369 mother-infant pairs participating in a randomized controlled trial of praziquantel given at 12–16 weeks’ gestation. We performed regression analysis to address our study objectives. In maternal peripheral blood, the concentrations of CXCL8, and TNF receptor I and II decreased from 12 to 32 weeks’ gestation, while IL-13 increased. Praziquantel treatment did not significantly alter the trajectory of the concentration of any of the cytokines examined. Hookworm infection was associated with elevated placental IL-1, CXCL8 and IFN-γ. The risk of small-for-gestational age increased with elevated IL-6, IL-10, and CXCL8 in cord blood. The risk of prematurity was increased when cord blood sTNFRI and placental IL-5 were elevated.

**Conclusions:**

Our study suggests that fetal cytokines, which may be related to infectious disease exposures, contribute to poor intrauterine growth. Additionally, hookworm infection influences cytokine concentrations at the maternal-fetal interface.

**Clinical Trial Registry number and website:**

ClinicalTrials.gov (NCT00486863).

## Author summary

Schistosomiasis is one of the most prevalent parasitic tropical diseases, and it is primarily treated with the drug praziquantel. This study examined the effects of praziquantel treatment for schistosomiasis and the presence of geohelminth infections during pregnancy on cytokines in maternal, placental, and cord blood, and examined the effects of pro-inflammatory signatures at the maternal-fetal interface on perinatal outcomes. We analyzed the data of 369 mother-infant pairs obtained from a randomized controlled trial of praziquantel given at 12–16 weeks’ gestation. Praziquantel treatment did not significantly alter the trajectory of the concentration of any of the cytokines examined. Elevated levels of both Th1 and Th2 cytokines were associated with the risk of adverse perinatal outcomes (small-for-gestational age and prematurity). Hookworm coinfection at 12 weeks’ gestation was, however, related to elevated levels of certain cytokines in the placenta (IL-1, IL-5, CXCL8 and IFN-γ).

## Introduction

Adverse perinatal outcomes account for a substantial proportion of the global burden of disease [1] and lay the foundation for health in later childhood, adolescence, and adulthood [2–5]. Low birthweight (LBW), fetal growth restriction (FGR) and preterm births together account for more than 80% of all neonatal deaths globally [6]. These conditions are more common in developing countries and a considerable part of this difference is attributable to poor nutrition and infections [6, 7]. Specifically, infections such as malaria are known to predispose to preterm births, FGR and fetal loss among offspring of affected pregnant women [8, 9].

With respect to helminthiasis, less is known with regard to treatment strategies for pregnant women. In a non-interventional study conducted in a *Schistosoma japonicum* endemic area, Kurtis and colleagues found increased concentrations of pro-inflammatory cytokines including interleukin-1 (IL-1β) and tumor necrosis factor (TNF) in placental and cord blood among women with *S*. *japonicum* infection [10]. Further, among infected women, that study found an increased risk for placental histopathologic evidence of an inflammatory response including acute subchorionitis. In a recent randomized controlled trial (RCT) however, Olveda and colleagues found that treatment with praziquantel at 12–16 weeks gestation had no impact on birthweight, or risk for LBW, small-for-gestational age (SGA), or prematurity [11]. This raised the concern that treatment during pregnancy may be too late to modify a pro-inflammatory response at the maternal-fetal interface (MFI).

Healthy pregnancies are characterized by a placental microenvironment that is biased toward a T-helper 2 (Th2) cytokine milieu [12, 13], and increased expression of pro-inflammatory cytokines in the placenta have been associated with poor pregnancy outcomes in both human and animal models [14–21]. Of particular relevance to pregnant women in low and middle-income countries (LMICs), studies have demonstrated that malaria alters the placental Th2 bias toward a pro-inflammatory microenvironment and is associated with poor pregnancy outcomes, particularly FGR [18, 19, 22]. Specifically, in human studies, increased placental TNF staining has been associated with increased risk of FGR in the context of malaria and lower birthweight in the context of schistosomiasis [10, 18]. Though alterations in placental cytokines likely contribute to both FGR and prematurity in the context of malaria and other infectious diseases of pregnancy, little is known about how helminth infections influence this environment and no studies have addressed whether treatment during pregnancy modifies this. A better understanding of these mechanisms could inform the timing of treatment for helminthiasis as well as its prioritization in the pre-natal period.

As part of the aforementioned RCT conducted in Leyte, The Philippines, we investigated whether treatment for schistosomiasis at 12–16 weeks’ gestation and the presence of other helminth infections would influence the cytokine micro-environment. Specifically, the objectives of this study were to 1) examine the impact of treatment with praziquantel on the inflammatory milieu in maternal, placental, and cord blood, 2) assess the extent to which proinflammatory signatures in placental and cord blood impacts the risk for LBW, SGA, and prematurity, and 3) evaluate the impact of other helminths on the inflammatory micro environment.

## Materials and methods

### Study design & population

This was a secondary analysis of data from a double blind placebo-controlled RCT examining the effects of praziquantel given at 12–16 weeks’ gestation for the treatment of schistosomiasis on pregnancy outcomes [11]. The RCT aimed to address the gaps in evidence concerning the safety and efficacy of praziquantel treatment, and thereby provision of praziquantel treatment to pregnant women infected with Schistosomiasis, in line with recommendations from the World Health Organization (WHO). Briefly, pregnant women presenting for prenatal care at six Municipal Health Centers servicing approximately 50 baranguays (villages) in a schistosomiasis endemic region of Leyte, The Philippines, were approached by midwives for screening. Initial eligibility screening included a urine pregnancy test and three stool samples collected on different days for the quantification of *S*. *japonicum* and soil transmitted helminths (STHs) eggs using the Kato-Katz method [23, 24]. The second phase of screening and enrollment was conducted at Remedios Trinidad Romualdez (RTR) Hospital in Tacloban, Leyte. The study physician performed a trans-abdominal ultrasound to assess fetal viability and estimate gestational age. Women were eligible if they provided informed consent and were infected with *S*. *japonicum*, age 18 or older, otherwise healthy as determined by physician history, physical examination and laboratory studies, and pregnant at 12–16 weeks’ gestation with a live, singleton, intrauterine fetus. Women who met eligibility criteria (n = 370) were randomly assigned (1:1) to receive either over-encapsulated praziquantel (30 mg/kg × 2) or over-encapsulated placebo (dextrose), as a split dose over three hours in a double-blind fashion.

### Baseline & follow-up

At 12–16 weeks’ gestation, a detailed demographic and medical history was collected and physical examination (including anthropometric measures) conducted. Weight, height and other anthropometric measures were made as described [25, 26]. Anthropometric measures were repeated at 32 weeks’ gestation. Venous blood samples were collected at 12-weeks and at 32-weeks gestation for assessment of inflammatory and hematologic biomarkers. Women were scheduled for additional visits as needed based on obstetrician-identified diagnoses. All women received prenatal vitamins with iron, as per standard of prenatal care in The Philippines.

Stool samples were collected and intensity of helminth infection was determined as the mean of the three samples, and categorized using WHO criteria as follows: *S*. *japonicum*, low, moderate and heavy intensity infections were defined as 1–99, 100–399 and ≥400 eggs per gram (epg), respectively; *Ascaris lumbricoides*, low, moderate and heavy intensity infections were defined as 1–4,999, 5,000–49,999 and ≥50,000 epg, respectively; *Trichuris trichuria*, low, moderate and heavy intensity infections were defined as 1–999, 1,000–9,999 and ≥10,000 epg, respectively; hookworm, low, moderate, and heavy intensity were defined as 1–1,999, 2,000–3,999 and ≥4,000 epg, respectively [23, 24].

### Delivery

Following initial stabilization of the newborn and mother, placental samples (wedge biopsy and pooled blood) and cord blood were collected. Newborns were examined and weighed within 48 hours of delivery on a Tanita model BD 585 portable scale (Arlington Heights, MD). LBW was defined as birthweight below 2500g, and SGA as birthweight below the 10^th^ percentile for gestational age based on the INTERGROWTH standard [27]. Preterm birth was defined as a birth before 37 weeks’ gestational age.

### Biomarker assessment

Maternal 12-week, 32-week, placental, and cord blood serum samples were aliquoted and stored at -80°C prior to testing. All available samples at each timepoint were used for comprehensive biomarker testing–only 238 cord blood samples were available. Assessment of biomarkers in the blood samples was conducted at the Center for International Health Research Laboratory in Providence, RI, USA. Biomarkers measured include IL-1, 2, 4, 5, 6, 8, 10, 12 and 13, interferon gamma (IFN-γ), TNF, chemokine ligand-9 (CXCL9) and soluble TNF receptors I and II (sTNFRI and sTNFRII). Analytes were quantified using a multiplex bead-based platform (Bio-Rad, Hercules, CA) as described previously [28]. The lower limit of detection was 2.44ng/L for most cytokines and 4.88ng/L for TNF receptors. Participants with undetectable concentrations of biomarkers were assigned the lowest detectable concentrations.

### Statistical analysis

In analyses examining the impact of praziquantel treatment and helminth infections on cytokine production in maternal, placental and cord blood, these biomarkers were outcome measures. These biomarkers were separately evaluated as predictors of adverse pregnancy outcomes. Cytokine production was considered as exposure or outcome in this analysis. Three different measures of cytokine production were also employed: (i) cytokine concentration in ng/L, (ii) the proportion of those with an 'elevated' cytokine concentration, and (iii) the proportion with cytokine present at a level above the assay detection limit. The means (±SE) of maternal cytokine concentrations at 12- and 32-weeks’ gestation were also estimated and the mean difference and 95% confidence interval (CI) estimated.

### Praziquantel treatment and cytokine production

To investigate the effect of praziquantel treatment on cytokine production, the proportions of participants with cytokine concentrations above detection limits in maternal 32 weeks’, placental, and cord blood samples were compared across treatment groups, and *P*-values obtained from Fisher’s exact tests. Further, the means (±SE) of cytokine concentrations at 32-weeks’ gestation (with 95% CI) were estimated within treatment subgroups and compared using linear regression. The extent to which the ratios of placental blood cytokines to maternal 12-week cytokines, and placental blood cytokines to maternal 32-week cytokines differed by treatment was also evaluated using Wilcoxon rank-sum tests.

### STH coinfection, cytokine production and perinatal outcomes

Generalized estimating equation regression models were used to assess the impact of each helminth infection at 12 weeks’ gestation on the proportion of participants with cytokines at a level above the assay detection limits in maternal 12- and 32-weeks’ gestation, placental and cord blood samples. Log-binomial models were used to evaluate the relationship between elevated maternal 32-week peripheral cytokines and placental and cord blood cytokines, and risk ratios (RRs) with 95% CI obtained. Log-binomial models were also used to examine the influence of elevated placental and cord blood cytokines on the risk of LBW, SGA, and prematurity. Log-binomial models provide RR estimates, which are intuitive and more appropriate for non-case control studies. The log-binomial model is however numerically unstable, and often fails to converge, and in those instances, log-Poisson models, which provide consistent but not fully efficient estimates of the RR and its CIs were employed [29].

### Adjustment for confounding

Potential confounders known to be related to cytokines and/or perinatal outcomes were considered for inclusion in multivariable models. In addition, potential confounders were identified through stepwise regression techniques, significant at *P*-value <0.15, with no variables forced into the model. Regression models were adjusted for predictors as specified in the footnotes of the respective tables and figures. Variables included in the models were praziquantel treatment, maternal age (<30 y, ≥30 y), newborn sex (boy, girl), maternal height (cm), maternal weight at 12 weeks (kg), maternal underweight (body mass index <18.5kg/m^2^), parity (number), socioeconomic status (quartiles), reported smoking status (yes, no), alcohol use (yes, no), and detection of *S*. *japonicum*, *A*. *lumbricoides*, *T*. *trichuria*, and hookworm, at 12 and 32 weeks’ gestation (yes, no).

### Effect modification

*P*-values for effect modification were obtained by introducing an interaction term to the log-binomial regression model, in which praziquantel treatment status was multiplied by the biomarker category, and the model compared to the model without the interaction term using the likelihood ratio test. Possible effect modification by hookworm infection at 12 weeks’ gestation was also explored.

### Statistical significance

*P*-values were 2-sided and statistical significance was defined as *P*-value <0.001, based on the Bonferroni correction for the familywise error rate (α /N, where *α* is 0.05 and *N* is the number of tests conducted in most of the analysis sets–N = 50), to account for multiple comparisons [30]. CIs were constructed at the 1-*α* level. All data in our study were de-identified. Analyses were conducted using SAS 9.4 (SAS Institute, Cary, NC).

### Ethics

The study was approved by both the Rhode Island Hospital Institutional Review Board in Providence, RI, USA and the Ethics Review Board of the Research Institute of Tropical Medicine in Manila, The Philippines. This trial was registered with ClinicalTrials.gov, number NCT00486863.

## Results

Participants included in this analysis were 369. Detailed information on the cohort’s participant characteristics have been previously presented [11]. Most of the infants in this cohort were born at term (median gestational age– 39 weeks (IQR: 38, 39), by vaginal delivery (341, 95%) and mean (±SD) birthweight was 2.85kg (±0.42). The prevalence of LBW, prematurity and SGA were 14% (n = 50), 9% (n = 32) and 23% (n = 83), respectively.

**Fig 1:**
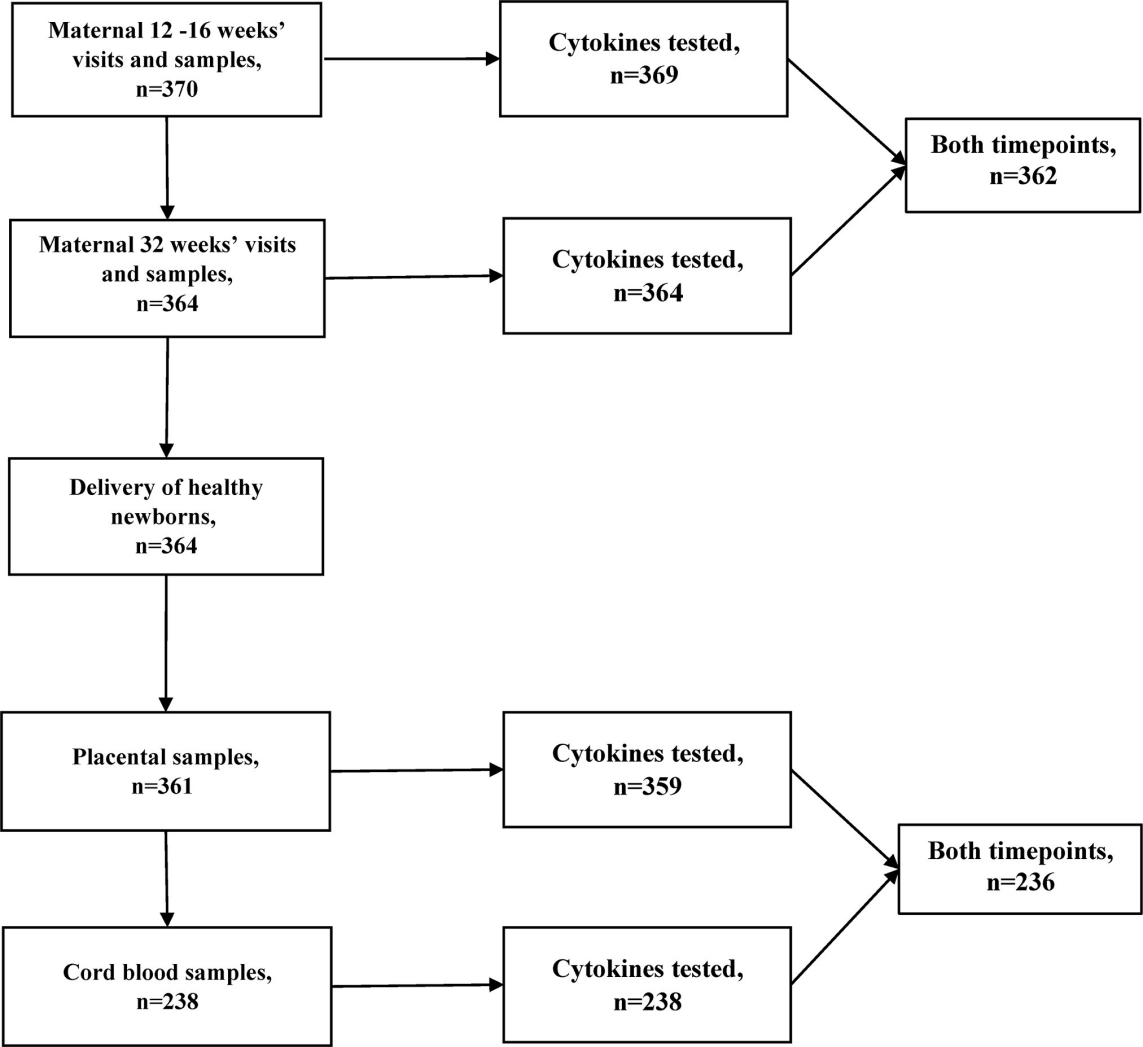
Flowchart of sample collection and biomarker testing.

**Fig 1** details the selection of samples for cytokine quantification. Maternal cytokine concentrations significantly decreased from 12 to 32 weeks’ gestation (**S2 Supporting Information**) for sTNFRI (Mean difference = -71.9; 95% CI: -104, -39.6, *P*-value<0.0001). The concentration of sTNFRII (Mean difference = -26.2; 95% CI: -44.2, -8.1, *P*-value = 0.005), IL-6 (Mean difference = -13.4; 95% CI: -23.6, -3.19, *P*-value = 0.01) and CXCL8 (Mean difference = -6.32; 95% CI: -11.6, -1.10, *P*-value = 0.02) decreased while the concentration of IL-13 (Mean difference = 0.33; 95% CI: 0.11, 0.55, *P*-value = 0.003) increased from 12 to 32 weeks’ gestation but the Bonferroni corrected *P*-values were not significant.

**Fig 2:**
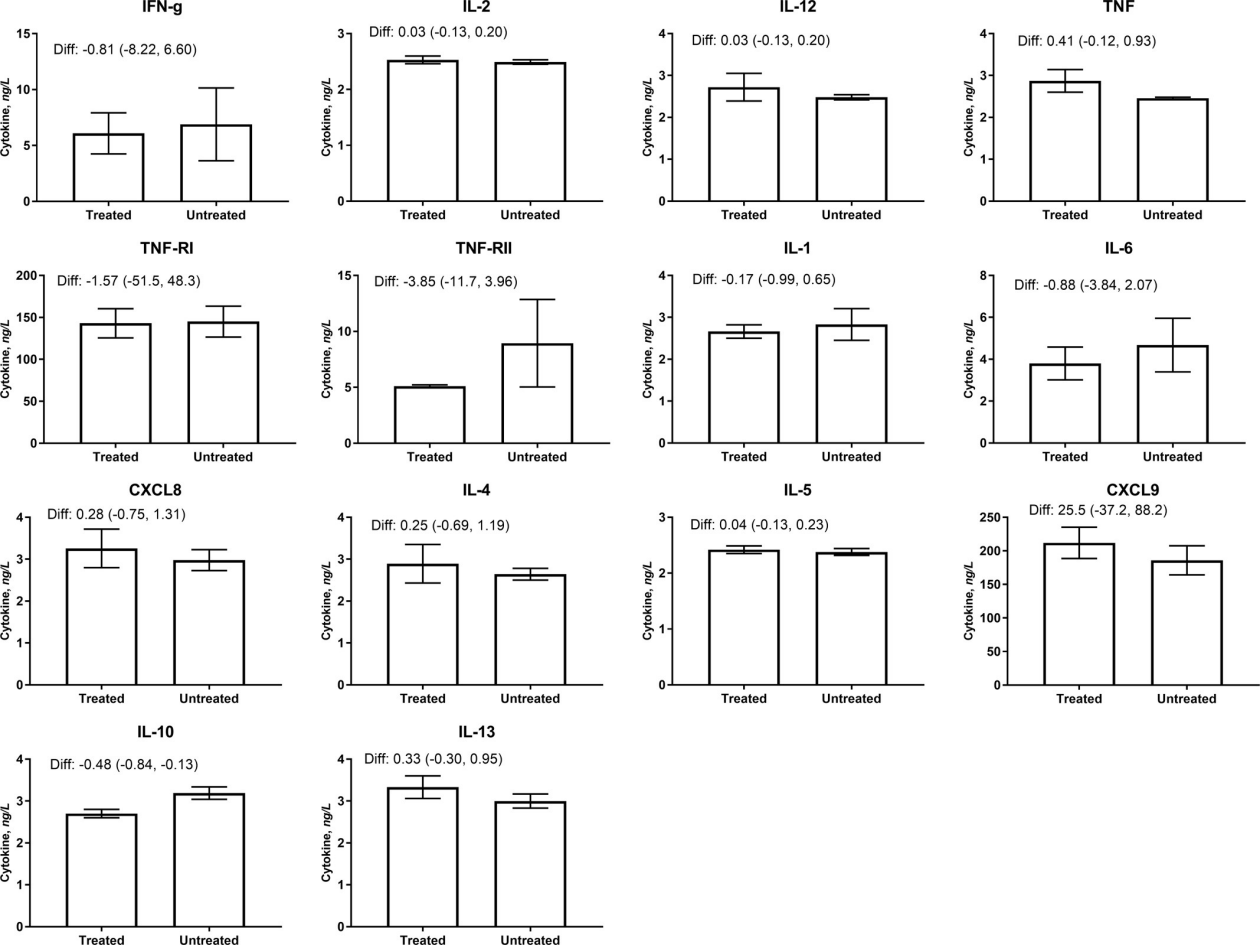
Maternal cytokine concentrations at 32 weeks' gestation, by praziquantel treatment status. Cytokine concentrations were modeled using linear regression models. Praziquantel treatment did not significantly modify cytokine concentrations.

The proportion of participants with detectable cytokines varied widely from 1–100% but tended to be highest in cord blood. To examine the impact of praziquantel treatment on cytokine concentrations, the concentration of cytokines in maternal blood at 32 weeks’ gestation was compared by treatment group (**Table 1 and Fig 2**). Praziquantel treatment lowered the concentration of anti-inflammatory IL-10 by 32-weeks’ gestation (Difference: -0.48 (-0.84, -0.13)), though the difference was not significant after Bonferroni’s correction (*P*-value = 0.008). Praziquantel treatment did not alter the concentration of other cytokines considerably. There was also no evidence that praziquantel significantly altered the likelihood of detecting cytokines in maternal serum at 12 and 32 weeks, or in placental or cord blood (**Table 2**).

**Table 1:** Maternal cytokine concentrations at 32 weeks’ gestation.

Cytokine type	Cytokine, ng/L	Treated, mean±SE (n=179)	Untreated, mean±SE (n=183)	Diff (95% CI)	*P*-value
Pro-inflammatory	IFN-γ	6.08±1.84	6.89±3.26	-0.81 (-8.22, 6.60)	0.83
	IL-2	2.53±0.07	2.49±0.04	0.03 (-0.13, 0.20)	0.68
	IL-12	2.72±0.33	2.48±0.06	0.25 (-0.40, 0.90)	0.45
	TNF	2.87±0.27	246±0.02	0.41 (-0.12, 0.93)	0.13
	sTNFRI	143.1±17.4	144.7±18.4	-1.58 (-51.5, 48.3)	0.95
	sTNFRII	5.09±0.13	8.94±3.91	-3.85 (-11.7, 3.96)	0.33
	IL-1	2.66±0.16	2.83±0.38	-0.17 (-0.99, 0.65)	0.68
	IL-6	3.80±0.78	4.68±1.28	-0.88 (-3.84, 2.07)	0.56
	CXCL8	3.26±0.46	2.98±0.25	0.28 (-0.75, 1.31)	0.59
Anti-inflammatory	IL-4	2.89±0.46	2.64±0.14	0.25 (-0.69, 1.19)	0.60
	IL-5	2.42±0.07	2.38±0.06	0.05 (-0.13, 0.23)	0.59
	CXCL9	211.5±23.3	186.0±21.7	25.5 (-37.2, 88.2)	0.42
	IL-10	2.70±0.10	3.19±0.15	-0.48 (-0.84, -0.13)	0.008
	IL-13	3.33±0.27	3.00±0.17	0.33 (-0.30, 0.95)	0.31

Means and SEs of maternal 32-week cytokine concentrations are presented by treatment group, and mean differences with 95% CI and *P*-values obtained using linear regression.

**Table 2:** Influence of praziquantel treatment on detection of inflammatory biomarker concentrations.

Cytokine type	Cytokine	Maternal blood at 32-weeks’ gestation, n = 362			Placental blood, n = 361			Cord blood, n = 238		
		Praziquantel, n = 179	Placebo, n = 183	*P*-value	Praziquantel, n = 177	Placebo, n = 184	*P*-value	Praziquantel, n = 121	Placebo, n = 117	*P*-value
Th1	IFN-γ	19 (11%)	13 (7%)	0.27	13 (7%)	11 (6%)	0.67	92 (76%)	91 (78%)	0.76
	IL-2	2 (1%)	2 (1%)	0.99	5 (3%)	2 (1%)	0.27	55 (45%)	62 (52%)	0.30
	IL-12	2 (1%)	7 (4%)	0.17	8 (5%)	5 (3%)	0.41	81 (67%)	74 (63%)	0.59
	TNF	7 (4%)	3 (2%)	0.22	34 (19%)	31 (17%)	0.58	72 (60%)	76 (65%)	0.42
	sTNFRI	179 (100%)	183 (100%)	0.99	177 (100%)	184 (100%)	0.99	121 (100%)	117 (100%)	0.99
	sTNFRII	179 (100%)	183 (100%)	0.99	176 (99%)	184 (100%)	0.49	121 (100%)	116 (99%)	0.25
Th2	IL-4	6 (3%)	5 (3%)	0.77	11 (6%)	10 (5%)	0.82	78 (64%)	75 (64%)	0.99
	IL-5	12 (7%)	11 (6%)	0.83	15 (9%)	12 (7%)	0.55	96 (79%)	99 (95%)	0.32
	CXCL9	134 (73%)	139 (78%)	0.33	132 (75%)	135 (73%)	0.72	108 (89%)	99 (85%)	0.34
	IL-10	48 (27%)	64 (35%)	0.11	66 (38%)	60 (33%)	0.32	100 (83%)	94 (80%)	0.74
	IL-13	39 (22%)	31 (17%)	0.24	47 (27%)	46 (25%)	0.72	87 (72%)	74 (63%)	0.17
Others	IL-1	2 (1%)	3 (1%)	0.99	25 (14%)	28 (15%)	0.88	77 (64%)	79 (68%)	0.59
	IL-6	8 (4%)	6 (3%)	0.59	94 (54%)	85 (46%)	0.17	91 (52%)	93 (54%)	0.67
	CXCL8	14 (8%)	13 (7%)	0.84	65 (37%)	70 (38%)	0.91	81 (67%)	85 (73%)	0.40
Ratio	IFN-γ:IL-4	19 (11%)	9 (5%)	0.04	18 (10%)	13 (7%)	0.35	12 (10%)	12 (10%)	0.99

Values are numbers of participants with detectable cytokine levels among those who received the respective treatment.

**Fig 3:**
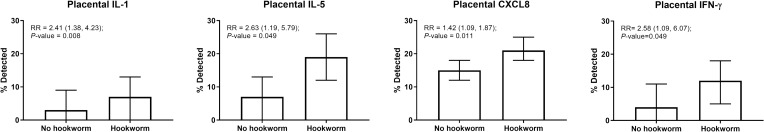
Relationship of elevated placental cytokines with coinfection with hookworm at 12 weeks' gestation. Cytokines were regarded as elevated if they exceeded the 90th percentile. *P*-values obtained from models adjusted for praziquantel treatment, fetal sex, maternal age, parity, underweight, and infection with any of *T*. *trichuria* and *A*. *lumbricoides* at 12 weeks' gestation. There were no other significant associations with other cytokines or with coinfection with *T*. *trichuria* or *A*. *lumbricoides*.

Although helminth infections were common at 12 weeks’ gestation (hookworm– 36%, *T*. *trichuria–* 81%, and *A*. *lumbricoides–* 62%), most were of light intensity (hookworm– 36%, *T*. *trichuria–* 73%, and *A*. *lumbricoides–* 28%). Hookworm infection was associated with a 1.42 to 2.58-fold increased risk of elevated placental levels above detection limits for some cytokines (**Fig 3**): IL-1 (RR = 2.41; 95% CI: 1.38, 4.23), IL-5 (RR = 2.63; 95% CI: 1.19, 5.79), CXCL8 (RR = 1.42, 95% CI: 1.09, 1.87) and IFN-γ (RR = 2.58; 95% CI: 1.09, 6.07) in multivariable models. Hookworm infection was not associated with an increased risk of detectable cytokines in maternal peripheral or cord blood (**S3 Supporting Information)**. Infection with *T*. *trichuria* and *A*. *lumbricoides* were also not associated with detectable levels in any of the cytokines (**S4 and S5 Supporting Informations)**. Hookworm infection at 12 weeks’ gestation did not modify the change in concentration from 12 to 32 weeks’ gestation.

We investigated the extent to which cytokine levels in maternal peripheral blood was related to cytokine levels in placental and cord blood in multivariable log-binomial regression models (**S6 Supporting Information**). **S7 Supporting Information** reports the concentration of each cytokine at which the 90^th^ percentile level was reached. Participants with elevated maternal 32-week IL-4 (RR = 17.3; 6.43, 46.4), IL-12 (RR = 14.2; 95% CI: 3.51, 57.1), and IFN-γ (RR = 5.35; 95% CI: 2.05, 14.0) were more likely to have elevated placental levels of the same cytokines. There were no significant associations in the level of maternal cytokines with the levels of the same cytokines in the cord blood.

**Fig 4:**
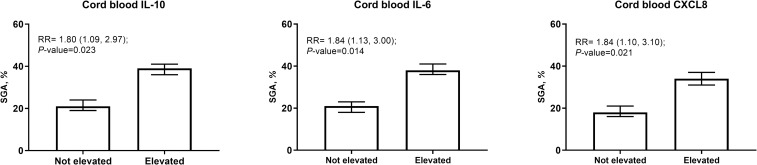
Relationship of elevated cord blood cytokines with SGA risk. Cytokines were regarded as elevated if they exceeded the 90^th^ percentile. *P* values obtained from models adjusted for praziquantel treatment, fetal sex, maternal age, parity, underweight, and infection with any of *T*. *trichuria*, *A*. *lumbricoides* and hookworm at 12 weeks' gestation.

**Fig 5:**
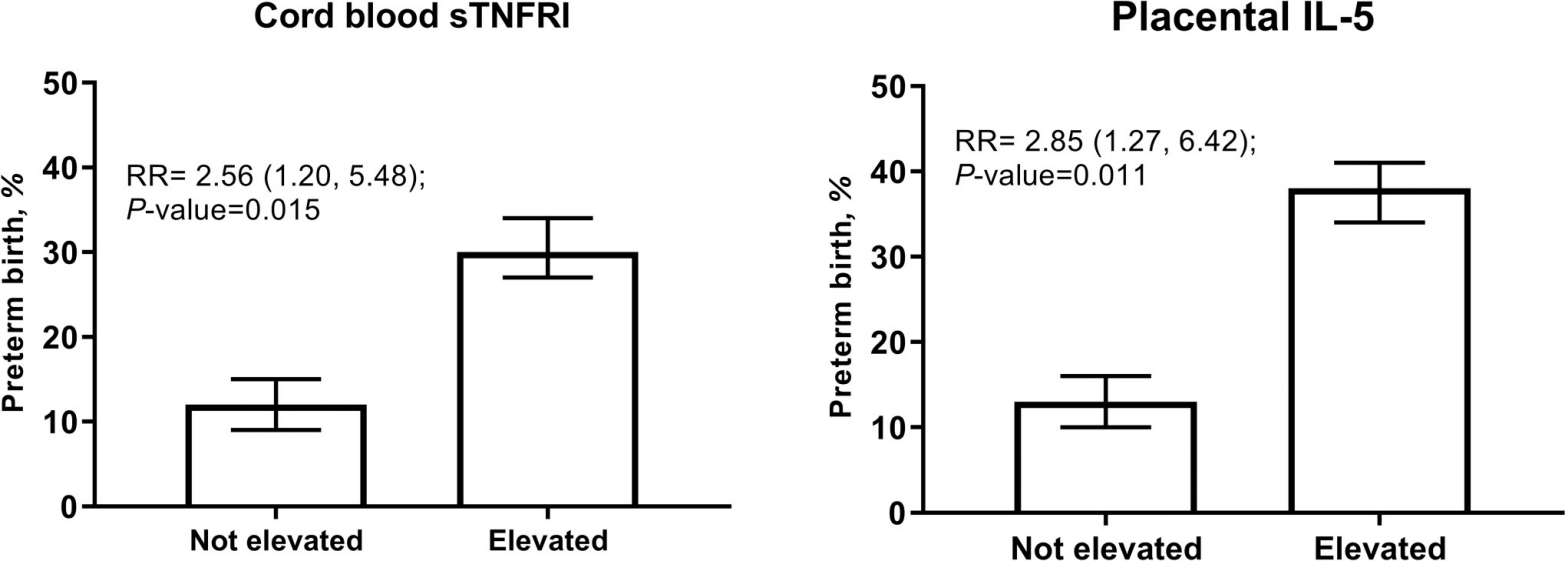
Relationship of cord blood sTNFRI and placental IL-5 with the risk of preterm birth. Cytokines were regarded as elevated if they exceeded the 90th percentile. *P*-values obtained from models adjusted for praziquantel treatment, fetal sex, maternal age, parity, underweight, and infection with any of *T*. *trichuria*, *A*. *lumbricoides* and hookworm at 12 weeks' gestation.

The prevalence of LBW, prematurity and SGA were 14% (n = 52), 9% (n = 33) and 23% (n = 84), respectively. Elevated levels of certain cytokines in the cord blood (**Tables 3 and 4)** were associated with 2-fold increased risk of SGA: IL-10 (Th2)–RR = 1.80 (1.09, 2.97), IL-6 –RR = 1.84 (1.13, 3.00), and CXCL8 –RR = 1.84 (1.10, 3.10) (**Fig 4**). Elevated sTNFRI (RR = 2.56; 95% CI: 1.20, 4.80) and IL-5 in placenta (RR = 2.85; 95% CI: 1.27, 6.42) were associated with increased risk of prematurity (**Fig 5**). These associations were not significant following Bonferroni correction. There was no evidence that levels of other placental and cord blood cytokines were related to the occurrence of prematurity, LBW and SGA.

**Table 3:** Association of elevated placental cytokines and birth outcomes (n = 361).

Placental cytokine	LBW			SGA			Preterm birth		
	n (%)	Adjusted RR (95% CI)	*P*-value	n (%)	Adjusted RR (95% CI)	*P*-value	n (%)	Adjusted RR (95% CI)	*P*-value
Pro-inflammatory									
IFN-γ	2 (9%)	0.53 (0.14, 2.01)	0.35	5 (22%)	0.86 (0.39, 1.89)	0.70	1 (4%)	0.43 (0.06, 3.02)	0.40
IL-2	1 (15%)	1.55 (0.24, 10.2)	0.64	3 (43%)	2.07 (0.83, 5.17)	0.12	0 (0%)	NA	NA
IL-12	2 (15%)	NA	NA	4 (31%)	1.14 (0.42, 3.07)	0.80	1 (8%)	NA	NA
TNF	5 (20%)	1.63 (0.71, 3.75)	0.25	8 (32%)	1.41 (0.78, 2.57)	0.26	2 (8%)	0.92 (0.23, 3.64)	0.90
sTNFRI	6 (17%)	1.23 (0.57, 2.64)	0.60	9 (26%)	1.12 (0.61, 2.03)	0.72	5 (14%)	1.67 (0.69, 4.02)	0.26
sTNFRII	7 (27%)	1.72 (0.87, 3.40)	0.12	6 (23%)	0.96 (0.47, 1.99)	0.92	3 (12%)	1.22 (0.40, 3.75)	0.73
IL-1	3 (11%)	0.70 (0.24, 2.07)	0.52	5 (19%)	0.74 (0.33, 1.66)	0.47	2 (7%)	0.72 (0.18, 2.87)	0.64
IL-6	2 (4%)	0.57 (0.15, 2.20)	0.42	7 (27%)	1.21 (0.62, 2.34)	0.58	1 (4%)	0.41 (0.06, 2.91)	0.37
CXCL8	3 (13%)	1.15 (0.38, 3.44)	0.80	4 (17%)	0.76 (0.30, 1.90)	0.56	1 (4%)	0.47 (0.07, 3.34)	0.45
Anti-inflammatory									
IL-4	3 (14%)	0.91 (0.31, 2.63)	0.86	6 (29%)	1.16 (0.58, 2.33)	0.67	1 (5%)	0.48 (0.07, 3.36)	0.46
IL-5	6 (22%)	1.53 (0.73, 3.20)	0.26	7 (26%)	1.08 (0.55, 2.09)	0.83	6 (22%)	2.85 (1.27, 6.42)	0.01
CXCL9	3 (18%)	NA	NA	3 (18%)	0.78 (0.27, 2.26)	0.65	2 (12%)	1.29 (0.33, 5.05)	0.71
IL-10	4 (15%)	1.09 (0.43, 2.75)	0.86	9 (33%)	1.42 (0.81, 2.49)	0.22	3 (11%)	1.26 (0.41, 3.88)	0.68
IL-13	3 (10%)	NA	NA	10 (34%)	1.54 (0.90, 2.62)	0.11	0 (0%)	NA	NA

n (%) represents the number of participants with LBW, SGA or preterm birth among the exposed (with elevated cytokine levels). Each log-binomial (or log-poisson) regression model was adjusted for praziquantel treatment, socioeconomic status, fetal sex, maternal age, parity, underweight, gestational age at birth, infection with any of *T*. *trichuria*, *A*. *lumbricoides* and hookworm at 12 weeks’ gestation, smoking and alcohol consumption. NA, not applicable.

**Table 4:** Association of elevated cord blood cytokines and birth outcomes (n = 238).

Cord blood cytokine	LBW			SGA			Preterm birth		
	n (%)	Adjusted RR (95% CI)	*P*-value	n (%)	Adjusted RR (95% CI)	*P*-value	n (%)	Adjusted RR (95% CI)	*P*-value
Pro-inflammatory									
IFN-γ	4 (22%)	2.08 (0.85, 5.13)	0.11	4 (22%)	0.92 (0.39, 2.19)	0.85	0 (0%)	NA	NA
IL-2	6 (27%)	2.43 (0.98, 6.04)	NA	9 (41%)	2.15 (0.61, 7.58)	0.23	0 (0%)	NA	NA
IL-12	0 (0%)	NA	NA	3 (19%)	0.74 (0.27, 2.04)	0.56	2 (13%)	2.00 (0.49, 8.11)	0.33
TNF	2 (13%)	0.85 (0.23, 3.13)	0.80	5 (33%)	1.49 (0.70, 3.16)	0.30	1 (7%)	0.78 (0.11, 5.49)	0.80
sTNFRI	6 (18%)	1.25 (0.58, 2.67)	0.57	7 (21%)	0.86 (0.43, 1.71)	0.67	7 (21%)	2.56 (1.20, 5.48)	0.02
sTNFRII	5 (15%)	1.01 (0.44, 2.35)	0.98	3 (9%)	0.37 (0.12, 1.09)	0.07	6 (18%)	2.11 (0.93, 4.80)	0.07
IL-1	2 (9%)	0.67 (0.17, 2.57)	0.56	7 (30%)	1.57 (0.78, 3.16)	0.21	2 (9%)	1.19 (0.29, 4.93)	0.82
IL-6	6 (18%)	1.38 (0.64, 2.99)	0.42	13 (38%)	1.84 (1.13, 3.00)	0.01	3 (9%)	0.97 (0.31, 3.03)	0.96
CXCL8	4 (15%)	1.12 (0.44, 2.85)	0.82	10 (38%)	1.84 (1.10, 3.10)	0.02	3 (12%)	1.71 (0.52, 5.57)	0.38
Anti-inflammatory									
IL-4	5 (22%)	1.85 (0.83, 4.12)	0.13	9 (39%)	1.39 (0.81, 2.40)	0.23	4 (17%)	2.93 (1.06, 8.10)	0.04
IL-5	3 (13%)	0.85 (0.29, 2.47)	0.76	7 (30%)	1.06 (0.56, 2.00)	0.87	3 (13%)	1.92 (0.60, 6.15)	0.27
CXCL9	3 (14%)	1.17 (0.41, 3.36)	0.77	8 (36%)	1.38 (0.79, 2.42)	0.26	1 (5%)	0.66 (0.09, 4.71)	0.68
IL-10	3 (14%)	1.17 (0.42, 3.30)	0.77	9 (43%)	1.80 (1.09, 2.97)	0.02	1 (5%)	0.72 (0.10, 5.15)	0.74
IL-13	2 (9%)	0.70 (0.18, 2.72)	0.61	6 (26%)	1.15 (0.57, 2.34)	0.69	15 (7%)	1.25 (0.31, 5.08)	0.75

n (%) represents the number of participants with LBW, SGA or preterm birth among the exposed (with elevated cytokine levels). Each log-binomial (or log-poisson) regression model was adjusted for praziquantel treatment, fetal sex, maternal age, parity, underweight, gestational age at birth, infection with any of *T*. *trichuria*, *A*. *lumbricoides* and hookworm at 12 weeks’ gestation, smoking and alcohol consumption. NA, not applicable.

We investigated effect modification of the association of cord and placental cytokines with the risk of perinatal outcomes by praziquantel treatment and hookworm infection at 12 weeks’ gestation and found no significant effect modification. We also examined the baseline characteristics of included and excluded participants and observed no significant differences in the characteristics of both groups with respect to the 12 weeks’, 32 weeks’ and placental analyses. Included mothers that contributed to the cord blood analyses were of slightly higher BMI and heavier hookworm egg burden at 12 weeks’ gestation compared to excluded participants (**S8 Supporting Information)**.

## Discussion

In a cohort of pregnant women in The Philippines infected with *S*. j*aponicum* and enrolled in a placebo-controlled RCT of praziquantel treatment, we examined the extent to which helminth coinfection and praziquantel treatment modified the cytokine milieu in the maternal, placental, and fetal compartments. We further investigated the relationship between the cytokine micro-environments and risk of adverse pregnancy outcomes. While praziquantel treatment did not alter the concentrations of the cytokines, hookworm infection was associated with higher levels of some placental cytokine. We also found that the concentrations of specific pro-inflammatory and anti-inflammatory cytokines in the placenta and cord blood were related to the risk of SGA and prematurity.

Evidence from animal and human studies suggests that maternal infections alter the placental and fetal inflammatory milieu, with important implications for health during the neonatal period and childhood [31–33]. For instance, Kurtis and colleagues have previously shown that maternal schistosomiasis is associated with a pro-inflammatory cytokine response in maternal, placental, and fetal compartments [10]. McDonald and colleagues have also demonstrated that schistosome egg antigens elicit pro-inflammatory immune responses from trophoblast cells in vitro, such that direct infection of the placenta may not be necessary to drive these responses [34]. In addition, McDonald and colleagues have found that infection with *S*. *japonicum* was associated with elevated endotoxin levels in placental blood and this was, in turn, associated with a pro-inflammatory signature [35]. It is thought that endotoxin is elevated in the context of schistosomiasis due to microbial translocation as eggs traverse the gut wall from the normally sterile systemic circulation into the gut lumen. In the context of malaria, altered placental cytokine concentrations have been demonstrated in the presence of infection, with increased expression of both pro-inflammatory cytokine (IL-1β and TNF) and chemokines (CXCL8), and decreased expression of IL-6 [18]. In this analysis, we also found that hookworm infection among pregnant women was associated with elevated Th1 (IL-1β and IFN-γ) cytokines, as well as IL-5 and CXCL8 in blood collected from the maternal-fetal interface.

Cytokine production at the maternal-fetal interface is crucial for many aspects of healthy pregnancy, including protection of the fetus from invading pathogens and the initiation of labor [36, 37]. Infiltration of leucocytes into the myometrium has been demonstrated in both term and preterm labor, with the type of cells and cytokine elevation patterns being dependent on the presence or type of specific immune triggers [38, 39]. We observed a 2-fold increased risk in preterm births in the presence of elevated cord blood sTNFRI, the soluble component of TNF receptor 2 through which TNF facilitates prostaglandin production to initiate uterine contractions [40, 41].

Altered cytokine production by the placenta may contribute to the risk of FGR, a process through which an adverse intrauterine environment places the newborn at risk for SGA birth [18, 42, 43]. In a previous study, Kurtis and colleagues had shown that placental blood IL-1β and TNFα were related to birthweight in a Filipino pregnancy cohort [10]. In the present study, pregnancies with elevated cord blood IL-10, IL-6 and CXCL8 each had about 2-fold greater risk of SGA after adjusting for multiple potential confounders. IL-1 and IL-6 are proinflammatory. IL-10 is anti-inflammatory and belongs to the Th2 subset [44]. CXCL8 is a neutrophil chemotactic and activating factor produced by monocytes, and trophoblasts in normal human pregnancy [45]. CXCL8 production increases during infections and in response to LPS and pro-inflammatory cytokines (TNF and IL-1) [46]. As part of the Th2 response, IL-13 inhibits the production of multiple cytokines including TNF, IL-10, and IL-1β [47]. Costimulation of Th2-associated cytokines to counteract the effects of pro-inflammatory Th1 cytokines during an infection likely explains the associations with SGA observed. A similar pattern has been previously reported among Tanzanian pregnant women with placental malaria where both pro-inflammatory CXCL9 and anti-inflammatory IL-10 were observed to be related risk for LBW [48].

Praziquantel treatment leads to a substantial and prolonged immune response due to the release of immunogenic antigens from dying eggs and worms, a decrease in T regulatory cells, and increased production of both Th1 and Th2 cytokines [49]. In this study, praziquantel treatment did not significantly alter the trajectory of the concentration of any of the cytokines examined. Our results differ from previous studies among non-pregnant individuals infected with *S*. *mansoni* that have reported increases in Th2 cytokines following praziquantel treatment [50–52]. It, however, remains possible that schistosomiasis infection alters the inflammatory milieu at the MFI, but the prolonged immune response to treatment does not allow modification of this milieu during gestation, suggesting active treatment of all women of reproductive age as recently recommended [53].

There are limitations to this study that should be addressed. First, all women had *S*. *japonicum* infection at study inception, somewhat limiting generalizability. Although placental blood using wedge biopsy leads to substantial contamination with maternal blood, our interest in understanding the broader cytokine milieu at the MFI and its impact on birth outcomes support this approach [54]. Further cytokine biology is complex, and phenomena such as co-stimulation, redundancy and synergy complicate the interpretation of findings, particularly the attribution of causality to specific cytokines in mediating adverse birth outcomes. Our study was conducted in a setting of multiple, often comorbid parasitic infections, limiting our ability to definitively attribute variations in cytokine concentrations to the presence or intensity of individual infections. Finally, cytokine profiles appear to differ by complex constructs linked to race [55], and this further limits the generalizability of our findings. We examined the associations of placental cytokines above the 90^th^ percentile with the risk of clinical outcomes, though we are unable to rule out the possibility that thresholds differ for each cytokine. Limited statistical power and measurement error may also some of the insignificant findings from our analysis. We also cannot rule out potential unmeasured cofounding in some of the analysis. Finally, we adjusted *P*-values for multiple testing due to the large number of statistical tests performed to reduce the possibility that our findings may be due to chance; however, the consistency of our results and how these are related to the extant literature further support their veracity.

We analyzed data from an RCT to examine the influence of alterations in the balance of cytokines during gestation on the risk of perinatal and neonatal outcomes. Our analysis examined intermediate steps in the causal pathway from praziquantel treatment to adverse pregnancy outcomes including FGR. Our finding of a lack of effect of praziquantel on cytokines is consistent with the main RCT’s null findings [11] with respect to FGR, in spite of significant associations of elevated cytokines and pregnancy outcomes. We found that hookworm coinfection among pregnant women with schistosomiasis was associated with elevated cytokine concentrations at the MFI, which is in turn associated with increased risk of FGR and preterm births. Our findings strengthen the evidence in favor of prenatal treatment of women of reproductive age group for both schistosomiasis and STHs.

## Supporting information

S1 Supporting Information CONSORT checklist. (DOC)

S2 Supporting Information Maternal cytokine concentrations at 12- and 32-weeks’ gestation. (DOCX)

S3 Supporting Information Influence of hookworm coinfection at 12 weeks’ gestation on detectable cytokine levels during pregnancy. (DOCX)

S4 Supporting Information Influence of *A. lumbricoides* coinfection at 12 weeks’ gestation on detectable cytokine levels during pregnancy. (DOCX)

S5 Supporting Information Influence of *T. trichuria* coinfection at 12 weeks’ gestation on detectable cytokine levels during pregnancy. (DOCX)

S6 Supporting Information Relationship of maternal 32-week cytokine levels with placental and cord blood cytokine concentrations. (DOCX)

S7 Supporting Information Cutoffs for elevated (>90th percentile) cytokines. (DOCX)

S8 Supporting Information Comparison of included and excluded participants’ characteristics. (DOCX)
