# Supplementary material for: Maternal, placental and cord blood cytokines and the risk of adverse birth outcomes among pregnant women infected with Schistosoma japonicum in the Philippines
Source: PLoS Negl Trop Dis. 2019 Jun 12;13(6):e0007371. doi: 10.1371/journal.pntd.0007371 (PMC6590831; doi:10.1371/journal.pntd.0007371)
Supplement: S2 Supporting Information — (DOCX) [file pntd.0007371.s002.docx]

S2 Supporting Table 1. Maternal cytokine concentrations at 12- and 32-weeks’ gestation (n=369)

| Cytokine type | Cytokine, ng/L | 12 weeks’ gestation,  Mean±SE | 32 weeks’ gestation,  Mean±SE | Change,  Mean (95% CI)^1^ | *P*-value |
| --- | --- | --- | --- | --- | --- |
| Th1 | IFN-γ | 6.96±1.50 | 6.49±1.88 | -0.31 (-3.39, 2.76) | 0.84 |
|  | IL-2 | 2.73±0.25 | 2.51±0.04 | -0.22 (-0.73, 0.28) | 0.38 |
|  | IL-12 | 2.81±0.30 | 2.59±0.16 | -0.13 (-0.32, 0.06) | 0.17 |
|  | TNF | 2.59±0.16 | 2.66±0.13 | 0.07 (-0.05, 0.19) | 0.25 |
|  | sTNFRI | 215.5±16.4 | 143.9±12.7 | -71.88 (-104.14, -39.63) | <.0001* |
|  | sTNFRII | 32.7±10.9 | 7.04±1.99 | -26.16 (-44.17, -8.14) | 0.005 |
| Th2 | IL-4 | 2.75±0.25 | 2.76±0.24 | 0.01 (-0.12, 0.14) | 0.87 |
|  | IL-5 | 2.56±0.17 | 2.40±0.04 | -0.16 (-0.53, 0.20) | 0.37 |
|  | CXCL9 | 214.1±28.4 | 198.6±15.9 | -17.03 (-78.63, 44.57) | 0.59 |
|  | IL-10 | 3.07±0.22 | 2.95±0.09 | -0.12 (-0.56, 0.33) | 0.60 |
|  | IL-13 | 2.82±0.11 | 3.16±0.16 | 0.33 (0.11, 0.55) | 0.003 |
| Others | IL-1 | 2.72±0.16 | 2.74±0.21 | 0.03 (-0.43, 0.49) | 0.91 |
|  | IL-6 | 17.4±5.10 | 4.24±0.75 | -13.4 (-23.6, -3.19) | 0.01 |
|  | CXCL8 | 9.31±2.58 | 3.12±0.26 | -6.32 (-11.6, -1.10) | 0.02 |

^1^ Negative values represent decreases in absolute cytokine concentrations while positive values represent increases. **P*-value for significance is set at 0.001, in accordance with Bonferroni’s correction for the familywise error rate.
