# Supplementary material for: Maternal, placental and cord blood cytokines and the risk of adverse birth outcomes among pregnant women infected with Schistosoma japonicum in the Philippines
Source: PLoS Negl Trop Dis. 2019 Jun 12;13(6):e0007371. doi: 10.1371/journal.pntd.0007371 (PMC6590831; doi:10.1371/journal.pntd.0007371)
Supplement: S3 Supporting Information — (DOCX) [file pntd.0007371.s003.docx]

S3 Supporting Table 2. Influence of hookworm coinfection at 12 weeks’ gestation on detectable cytokine levels during pregnancy

| Cytokine type | Cytokine | Maternal blood at 12-weeks’ gestation | | | Maternal blood at 32-weeks’ gestation | | | Placental blood | | | Cord blood | | |
| --- | --- | --- | --- | --- | --- | --- | --- | --- | --- | --- | --- | --- | --- |
|  |  | n (%) | Adjusted  RR (95% CI) | *P*-value | n (%) | Adjusted  RR (95% CI) | *P*-value | n (%) | Adjusted  RR (95% CI) | *P*-value | n (%) | Adjusted  RR (95% CI) | *P*-value |
| Pro-inflammatory | IFN-γ | 8 (9%) | 1.91 (0.71, 5.14) | 0.20 | 11 (11%) | 0.99 (0.49, 2.02) | 0.98 | 13 (13%) | 2.31 (1.01, 5.33) | 0.049 | 67 (75%) | 0.91 (0.67, 1.23) | 0.53 |
|  | IL-2 | 4 (5%) | 3.07 (0.52, 18.1) | 0.22 | 1 (1%) | NA | NA | 5 (5%) | NA | NA | 45 (51%) | 0.93 (0.71, 1.21) | 0.58 |
|  | IL-12 | 4 (5%) | 2.01 (0.52, 7.72) | 0.31 | 1 (1%) | 0.35 (0.04, 3.12) | 0.35 | 6 (6%) | 2.32 (0.67, 8.08) | 0.19 | 55 (62%) | 0.89 (0.63, 1.26) | 0.52 |
|  | TNF | 2 (2%) | 0.47 (0.10, 2.11) | 0.32 | 4 (4%) | 1.32 (0.36, 4.79) | 0.67 | 27 (27%) | 1.49 (0.95, 2.34) | 0.54 | 59 (66%) | 1.05 (0.87, 1.28) | 0.61 |
|  | sTNFRI | 130 (100%) | NA | NA | 130 (100%) | NA | NA | 128 (100%) | NA | NA | 128 (100%) | NA | NA |
|  | sTNFRII | 130 (100%) | NA | NA | 130 (100%) | NA | NA | 128 (100%) | NA | NA | 128 (100% | NA | NA |
|  | IL-1 | 4 (5%) | 1.31 (0.36, 4.73) | 0.68 | 1 (1%) | 0.59 (0.06, 6.03) | 0.66 | 25 (25%) | 2.19 (1.27, 3.76) | 0.005 | 60 (67%) | 1.01 (0.84, 1.22) | 0.89 |
|  | IL-6 | 13 (10%) | 1.470 (0.71, 3.06) | 0.29 | 7 (5%) | 1.72 (0.57, 5.14) | 0.33 | 65 (66%) | 1.18 (0.95, 1.47) | 0.14 | 66 (52%) | 0.99 (0.80, 1.23) | 0.95 |
|  | CXCL8 | 22 (25%) | 0.92 (0.59, 1.42) | 0.71 | 9 (9%) | 1.32 (0.57, 3.05) | 0.51 | 52 (53%) | 1.52 (1.15, 2.03) | 0.044 | 61 (69%) | 1.01 (0.84, 1.21) | 0.95 |
| Anti-inflammatory | IL-4 | 4 (5%) | 1.60 (0.44, 5.96) | 0.48 | 3 (3%) | 1.24 (0.26, 5.87) | 0.78 | 11 (11%) | 2.34 (0.92, 6.00) | 0.08 | 57 (64%) | 0.91 (0.65, 1.28) | 0.60 |
|  | IL-5 | 7 (8%) | 1.05 (0.44, 2.50) | 0.91 | 4 (4%) | 0.59 (0.19, 1.78) | 0.34 | 11 (11%) | 1.88 (0.84, 4.23) | 0.13 | 76 (85%) | 1.07 (0.94, 1.21) | 0.30 |
|  | CXCL9 | 88 (100%) | 1.01 (0.78, 1.30) | 0.96 | 95 (97%) | 0.98 (0.76, 1.27) | 0.88 | 98 (99%) | 0.99 (0.76, 1.27) | 0.92 | 82 (92%) | 0.99 (0.92, 1.08) | 0.93 |
|  | IL-10 | 24 (27%) | 0.99 (0.66, 1.51) | 0.99 | 32 (33%) | 1.14 (0.80, 1.64) | 0.47 | 42 (42%) | 1.03 (0.77, 1.39) | 0.82 | 79 (89%) | 1.13 (1.01, 1.27) | 0.036 |
|  | IL-13 | 11 (13%) | 1.46 (0.71, 2) | 0.31 | 25 (26%) | 1.39 (0.82, 2.04) | 0.27 | 28 (28%) | 1.14 (0.76, 1.71) | 0.47 | 60 (67%) | 0.97 (0.81, 1.16) | 0.72 |

n (%) represents the number of participants with detectable cytokine levels among those with hookworm coinfection at 12 weeks’ gestation. Each log-binomial (or log-poisson) regression model was adjusted for praziquantel treatment, socioeconomic status, fetal sex, maternal age, parity, underweight, gestational age at birth, infection with any of *T. trichuria,* *A. lumbricoides* and hookworm at 12 weeks’ gestation, smoking and alcohol consumption. NA, not applicable.
