# Supplementary material for: Maternal, placental and cord blood cytokines and the risk of adverse birth outcomes among pregnant women infected with Schistosoma japonicum in the Philippines
Source: PLoS Negl Trop Dis. 2019 Jun 12;13(6):e0007371. doi: 10.1371/journal.pntd.0007371 (PMC6590831; doi:10.1371/journal.pntd.0007371)
Supplement: S4 Supporting Information — (DOCX) [file pntd.0007371.s004.docx]

S4 Supporting Table 3. Influence of *A. lumbricoides* coinfection at 12 weeks’ gestation on detectable cytokine levels during pregnancy

| Cytokine type | Cytokine | Maternal blood at 12-weeks’ gestation | | | Maternal blood at 32-weeks’ gestation | | | Placental blood | | | Cord blood | | |
| --- | --- | --- | --- | --- | --- | --- | --- | --- | --- | --- | --- | --- | --- |
|  |  | n (%) | Adjusted  RR (95% CI) | *P*-value | n (%) | Adjusted  RR (95% CI) | *P*-value | n (%) | Adjusted  RR (95% CI) | *P*-value | n (%) | Adjusted  RR (95% CI) | *P*-value |
| Pro-inflammatory | IFN-γ | 15 (7%) | 1.33 (0.51, 3.46) | 0.56 | 16 (7%) | 0.58 (0.29, 1.16) | 0.12 | 13 (6%) | 0.80 (0.33, 1.91) | 0.61 | 114 (79%) | 1/10 (0.94, 1.28) | 0.23 |
|  | IL-2 | 5 (2%) | 3.54 (0.24, 36) | 0.29 | 4 (2%) | NA | NA | 4 (2%) | 1.09 (0.81, 1.41) | 0.64 | 70 (49%) | 1.07 (0.81, 1.41) | 0.64 |
|  | IL-12 | 5 (2%) | 0.89 (0.22, 3.53) | 0.87 | 6 (3%) | 1.25 (0.29, 5.34) | 0.76 | 7 (3%) | 0.88 (0.28, 2.79) | 0.83 | 96 (67%) | 1.04 (0.92, 1.18) | 0.53 |
|  | TNF | 9 (4%) | 1.04 (0.28, 3.85) | 0.96 | 6 (3%) | 1.04 (0.28. 3.85) | 0.96 | 40 (19%) | 1.09 (0.67, 1.75) | 0.73 | 90 (63%) | 1.12 (0.89, 1.40) | 0.35 |
|  | sTNFRI | 221 (100%) | 1.00 (0.80, 1.24) | 0.99 | 221 (100%) | 1.00 (0.80, 1.25) | 0.99 | 217 (100%) | 1.00 (0.80, 1.25) | 0.99 | 212 (100%) | 1.00 (0.80, 1.26 | 0.99 |
|  | sTNFRII | 221 (100%) | 1.00 (0.80, 1.24) | 0.99 | 221 (100%) | 1.00 (0.80, 1.25) | 0.99 | 217 (100%) | 1.01 (0.81, 1.26) | 0.95 | 212 (100%) | 1.02 (0.81, 1.28) | 0.89 |
|  | IL-1 | 4 (2%) | 0.54 (0.14, 2.08) | 0.37 | 2 (1%) | 0.45 (0.06, 3.47) | 0.44 | 33 (15%) | 1.25 (0.69, 2.29) | 0.46 | 93 (65%) | 1.06 (0.86, 1.31) | 0.56 |
|  | IL-6 | 20 (9%) | 1.43 (0.64, 3.17) | 0.38 | 11 (5%) | 2.31 (0.60, 8.91) | 0.23 | 113 (52%) | 1.04 (0.83, 1.30) | 0.75 | 109 (51%) | 0.95 (0.77, 1.18) | 0.66 |
|  | CXCL8 | 52 (24%) | 0.87 (0.60, 1.26) | 0.46 | 14 (6%) | 0.70 (0.31, 1.58) | 0.39 | 87 (40%) | 1.18 (0.87, 1.60) | 0.28 | 99 (69%) | 1.05 (0.86, 1.29) | 0.62 |
| Anti-inflammatory | IL-4 | 6 (3%) | 0.51 (0.15, 1.68) | 0.29 | 5 (2%) | 0.51 (0.15, 1.68) | 0.27 | 11 (5%) | 0.63 (0.26, 1.53) | 0.31 | 94 (65%) | 1.15 (0.93, 1.41) | 0.19 |
|  | IL-5 | 16 (7%) | 0.82 (0.37, 1.79) | 0.61 | 17 (8%) | NA | NA | 17 (8%) | 1.34 (0.58, 3.10) | 0.50 | 119 (83%) | 1.01 (0.74, 1.37) | 0.95 |
|  | CXCL9 | 159 (72%) | 1.02 (0.88, 1.18) | 0.81 | 161 (74%) | 0.93 (0.82, 1.07) | 0.31 | 165 (76%) | 1.09 (0.94, 1.26) | 0.24 | 128 (89%) | 1.04 (0.94, 1.16) | 0.42 |
|  | IL-10 | 48 (22%) | 0.70 (0.49, 1.01) | 0.05 | 66 (30%) | 0.98 (0.72, 1.35) | 0.91 | 72 (33%) | 0.91 (0.68, 1.21) | 0.51 | 120 (83%) | 1.03 (0.90, 1.19) | 0.64 |
|  | IL-13 | 13 (6%) | 0.46 (0.22, 0.93) | 0.03 | 42 (19%) | 0.90 (0.57, 1.41) | 0.65 | 55 (25%) | 1.01 (0.70. 1.47) | 0.95 | 101 (70%) | 1.04 (0.93, 1.16) | 0.48 |

n (%) represents the number of participants with detectable cytokine levels among those with hookworm coinfection at 12 weeks’ gestation. Each log-binomial (or log-poisson) regression model was adjusted for praziquantel treatment, socioeconomic status, fetal sex, maternal age, parity, underweight, gestational age at birth, infection with any of *T. trichuria,* *A. lumbricoides* and hookworm at 12 weeks’ gestation, smoking and alcohol consumption. NA, not applicable.
