# Supplementary material for: Maternal, placental and cord blood cytokines and the risk of adverse birth outcomes among pregnant women infected with Schistosoma japonicum in the Philippines
Source: PLoS Negl Trop Dis. 2019 Jun 12;13(6):e0007371. doi: 10.1371/journal.pntd.0007371 (PMC6590831; doi:10.1371/journal.pntd.0007371)
Supplement: S5 Supporting Information — (DOCX) [file pntd.0007371.s005.docx]

S5 Supporting Table 4. Influence of *T. trichuria* coinfection at 12 weeks’ gestation on detectable cytokine levels during pregnancy

| Cytokine  type | Cytokine | Maternal blood  at 12-weeks’ gestation | | | Maternal blood  at 32-weeks’ gestation | | | Placental blood | | | Cord blood | | |
| --- | --- | --- | --- | --- | --- | --- | --- | --- | --- | --- | --- | --- | --- |
|  |  | n (%) | Adjusted  RR (95% CI) | *P*-value | n (%) | Adjusted  RR (95% CI) | *P*-value | n (%) | Adjusted  RR (95% CI) | *P*-value | n (%) | Adjusted  RR (95% CI) | *P*-value |
| Pro-inflammatory | IFN-γ | 19 (7%) | 1.56 (0.45, 5.48) | 0.48 | 28 (10%) | 1.88 (0.67, 5.28) | 0.23 | 21 (7%) | 2.24 (0.51, 9.86) | 0.28 | 143 (76%) | 0.94 (0.80, 1.10) | 0.43 |
|  | IL-2 | 6 (2%) | NA | NA | 4 (1%) | NA | NA | 7 (2%) | NA | NA | 95 (50%) | 1.10 (0.78, 1.56) | 0.57 |
|  | IL-12 | 9 (3%) | NA | NA | 7 (2%) | NA | NA | 11 (4%) | 1.23 (0.26, 5.83) | 0.79 | 120 (63%) | 0.96 (0.83, 1.10) | 0.54 |
|  | TNF | 13 (4%) | 3.73 (0.47, 29.7) | 0.21 | 8 (3%) | NA | NA | 55 (19%) | 1.81 (0.87, 3.77) | 0.11 | 116 (61%) | 0.98 (0.77, 1.24) | 0.86 |
|  | TNF-RI | 293 (100%) | NA | NA | 291 (100%) | 1.00 (0.76, 1.31) | 0.99 | 289 (100%) | 1.00 (0.76, 1.31) | 0.99 | 281 (100%) | 1.00 (0.76, 1.31) | 0.99 |
|  | TNF-RII | 293 (100%) | 1.00 (0.76, 1.31) | 0.99 | 291 (100%) | 1.00 (0.76, 1.31) | 0.99 | 288 (99%) | 1.00 (0.76, 1.31) | 0.97 | 279 (100%) | 0.99 (0.75, 1.30) | 0.93 |
|  | IL-1 | 8 (3%) | NA | NA | 4 (1%) | 2.32 (0.18, 30.8) | 0.52 | 42 (15%) | 1.02 (0.49, 2.13) | 0.95 | 124 (66%) | 1.01 (0.80, 1.28) | 0.91 |
|  | IL-6 | 25 (9%) | 1.48 (0.52, 4.20) | 0.46 | 12 (4%) | 1.16 (0.25, 5.37) | 0.85 | 149 (52%) | 1.30 (0.96, 1.74) | 0.09 | 143 (51%) | 0.88 (0.61, 1.26) | 0.48 |
|  | CXCL8 | 74 (25%) | 1.13 (0.72, 1.79) | 0.60 | 18 (6%) | 0.49 (0.21, 1.12) | 0.09 | 112 (39%) | 1.30 (0.88, 1.94) | 0.19 | 132 (70%) | 1.01 (0.81, 1.26) | 0.93 |
| Anti-inflammatory | IL-4 | 9 (3%) | 2.17 (0.26, 17.8) | 0.47 | 10 (3%) | 2.18 (0.27, 17.4) | 0.46 | 19 (7%) | 2.22 (0.51, 9.67) | 0.29 | 119 (63%) | 0.88 (0.69, 1.12) | 0.31 |
|  | IL-5 | 25 (9%) | 2.21 (0.64, 7.57) | 0.21 | 19 (7%) | 0.97 (0.31, 2.98) |  | 19 (7%) | 0.51 (0.22, 1.19) | 0.12 | 157 (83%) | 0.89 (0.70, 1.11) | 0.30 |
|  | CXCL9 | 211 (72%) | 1.02 (0.85, 1.22) | 0.82 | 220 (76%) | 1.04 (0.88, 1.23) | 0.64 | 209 (73%) | 0.86 (0.75, 0.98) | 0.03 | 166 (88%) | 1.03 (0.90, 1.18) | 0.70 |
|  | IL-10 | 76 (26%) | 1.24 (0.76, 1.99) | 0.39 | 83 (29%) | 0.71 (0.51, 1.01) | 0.06 | 104 (36%) | 1.15 (0.77, 1.71) | 0.50 | 156 (83%) | 1.05 (0.87, 1.27) | 0.62 |
|  | IL-13 | 24 (8%) | 0.97 (0.43, 2.18) | 0.93 | 57 (20%) | 1.08 (0.61, 1.92) | 0.80 | 74 (26%) | 0.98 (0.62, 1.54) | 0.92 | 131 (69%) | 1.04 (0.91, 1.19) | 0.52 |

n (%) represents the number of participants with detectable cytokine levels among those with hookworm coinfection at 12 weeks’ gestation. Each log-binomial (or log-poisson) regression model was adjusted for praziquantel treatment, socioeconomic status, fetal sex, maternal age, parity, underweight, gestational age at birth, infection with any of *T. trichuria,* *A. lumbricoides* and hookworm at 12 weeks’ gestation, smoking and alcohol consumption. NA, not applicable.
