# Supplementary material for: Maternal, placental and cord blood cytokines and the risk of adverse birth outcomes among pregnant women infected with Schistosoma japonicum in the Philippines
Source: PLoS Negl Trop Dis. 2019 Jun 12;13(6):e0007371. doi: 10.1371/journal.pntd.0007371 (PMC6590831; doi:10.1371/journal.pntd.0007371)
Supplement: S6 Supporting Information — (DOCX) [file pntd.0007371.s006.docx]

**S6 Supporting Table 5.** Relationship of maternal 32-week cytokine levels with placental and cord blood cytokine concentrations

| Cytokine type | Cytokine | Maternal on placental cytokines, n=3541 | | | | Maternal on cord blood cytokines, n=236 | | | |
| --- | --- | --- | --- | --- | --- | --- | --- | --- | --- |
|  |  | Elevated Maternal,  n (%) | Elevated placental,  n (%) | Adjusted  RR  (95% CI)4^,^5 | *P*-value | Elevated Maternal,  n (%) | Elevated placental,  n (%) | Adjusted  RR  (95% CI)4^,^5 | *P*-value |
| Th1 | IFN-γ | 31 (9%) | 24 (7%) | 5.35  (2.05, 14.0) | 0.0006* | 21 (9%) | 182 (77%) | 1.11  (0.81, 1.52) | 0.53 |
|  | IL-2 | 1 (1%) | 7 (2%) | 20.7  (2.25, 190) | 0.007 | 4 (2%) | 22 (9%) | 2.37  (0.30, 18.9) | 0.42 |
|  | IL-12 | 9 (3%) | 13 (4%) | 14.2  (3.51, 57.1) | 0.0002* | 7 (3%) | 154 (65%) | 0.90  (0.61, 1.35) | 0.63 |
|  | TNF | 9 (3%) | 64 (18%) | 2.79  (1.06, 7.34) | 0.04 | 6 (3%) | 15 (6%) | 3.75  (0.38, 36.6) | 0.26 |
|  | sTNFRI | 35 (10%) | 35 (10%) | 0.27  (0.04, 2.02) | 0.20 | 33 (10%) | 33 (10%) | 2.08  (0.86, 5.08) | 0.11 |
|  | sTNFRII | 5 (1%) | 25 (7%) | 3.10  (0.37, 26.3) | 0.30 | 5 (1%) | 32 (9%) | NA | NA |
| Th2 | IL-4 | 11 (3%) | 21 (6%) | 17.3  (6.43, 46.4) | <.0001 | 4 (2%) | 193 (82%) | 2.56  (0.28, 23.1) | 0.40 |
|  | IL-5 | 22 (6%) | 25 (7%) | 1.06  (0.22, 5.07) | 0.94` | 4 (2%) | 193 (82%) | 0.74  (0.36, 1.49) | 0.40 |
|  | CXCL9 | 21 (6%) | 263 (74%) | 1.03  (0.87, 1.18) | 0.84 | 14 (6%) | 22 (9%) | 0.61  (0.08, 4.74) | 0.64 |
|  | IL-10 | 109 (31%) | 27 (8%) | 2.17  (0.97, 4.83) | 0.06 | 75 (32%) | 21 (9%) | 2.03  (0.80, 5.14) | 0.14 |
|  | IL-13 | 69 (19%) | 92 (26%) | 1.39  (0.91, 2.12) | 0.12 | 49 (21%) | 23 (10%) | 2.26  (0.93, 5.51) | 0.07 |
| Others | IL-1 | 5 (1%) | 28 (8%) | 8.46  (2.00, 35.8) | 0.004 | 1 (0.4%) | 155 (66%) | 1.69  (0.22, 13.2) | 0.61 |
|  | IL-6 | 13 (4%) | 176 (49%) | 1.41  (0.85, 2.34) | 0.19 | 13 (4%) | 34 (10%) | 1.56  (0.35, 6.90) | 0.56 |
|  | CXCL8 | 26 (7%) | 26 (7%) | 0.67  (0.09, 5.16) | 0.70 | 16 (7%) | 164 (69%) | 0.67  (0.41, 1.11) | 0.12 |
| Ratio | IFN-γ:IL-4 | 18 (9%) | 21 (10%) | 2.58  (0.93, 7.16) | 0.07 | 15 (9%) | 13 (8%) | 1.82  (0.53, 6.25) | 0.34 |

1 There were 354 individuals with both placental and maternal 32-week cytokine measures. 2 There were 236 individuals with both cord blood and maternal 32-week cytokine measures. 3n (%) represents the number of participants with elevated placental or cord blood cytokines among those with elevated maternal cytokines. 4 Values in the column are relative risks (RR) with 95% confidence intervals, and were obtained from log-binomial regression models. RR > 1 implies the outcome is more likely to occur when the exposure is present. RR < 1 implies the outcome is less likely when the exposure is present. 5Regression models were adjusted for praziquantel treatment, fetal sex, maternal age, parity, underweight, infection with any of hookworm, trichuris and ascaris at 12 weeks’ gestation, smoking and alcohol consumption. *P*-value for significance is set at 0.001, in accordance with Bonferroni’s correction for the familywise error rate.
