# Supplementary material for: Maternal, placental and cord blood cytokines and the risk of adverse birth outcomes among pregnant women infected with Schistosoma japonicum in the Philippines
Source: PLoS Negl Trop Dis. 2019 Jun 12;13(6):e0007371. doi: 10.1371/journal.pntd.0007371 (PMC6590831; doi:10.1371/journal.pntd.0007371)
Supplement: S7 Supporting Information — (DOCX) [file pntd.0007371.s007.docx]

S7 Supporting Table 6**.** Cutoffs for elevated (>90^th^ percentile) cytokines

| Cytokine type | Cytokine, ng/L | Maternal blood  at 12-weeks’ gestation,  n=267 | Maternal blood  at 32-weeks’ gestation,  n=276 | Placenta blood,  n=268 | Cord blood,  n=224 |
| --- | --- | --- | --- | --- | --- |
| Th1 | IFN-γ | 2.44 | 3.34 | 2.44 | 726.5 |
|  | IL-2 | 2.44 | 2.44 | 2.44 | 33.1 |
|  | IL-12 | 2.44 | 2.44 | 2.44 | 9.28 |
|  | TNF | 2.44 | 2.44 | 8.44 | 9.64 |
|  | sTNFRI | 541.3 | 444.0 | 4438 | 1855 |
|  | sTNFRII | 60.5 | 4.88 | 19.5 | 197.3 |
| Th2 | IL-4 | 2.44 | 2.44 | 2.44 | 9.18 |
|  | IL-5 | 2.44 | 2.44 | 2.44 | 9.5 |
|  | CXCL9 | 685.9 | 714.8 | 813.4 | 875.2 |
|  | IL-10 | 5.92 | 4.56 | 8.19 | 10.6 |
|  | IL-13 | 2.56 | 6.37 | 7.01 | 8.82 |
| Others | IL-1 | 2.44 | 2.44 | 7.80 | 53.1 |
|  | IL-6 | 2.44 | 2.44 | 350.0 | 435.7 |
|  | CXCL8 | 9.11 | 2.44 | 71.2 | 72.1 |
