# Supplementary material for: Maternal, placental and cord blood cytokines and the risk of adverse birth outcomes among pregnant women infected with Schistosoma japonicum in the Philippines
Source: PLoS Negl Trop Dis. 2019 Jun 12;13(6):e0007371. doi: 10.1371/journal.pntd.0007371 (PMC6590831; doi:10.1371/journal.pntd.0007371)
Supplement: S8 Supporting Information — (DOCX) [file pntd.0007371.s008.docx]

S8 Supporting Table 7**.** Comparison of included and excluded participants’ characteristics

| Variable  (median, IQR) | Maternal blood  at 12-weeks’ gestation | | | Maternal blood  at 32-weeks’ gestation | | | Placental blood | | | Cord blood | | |
| --- | --- | --- | --- | --- | --- | --- | --- | --- | --- | --- | --- | --- |
|  | Included,  n=267 | Excluded,  n=97 | *P*-value | Included  , n=276 | Excluded  , n=88 | *P*-value | Included,  n=268 | Excluded, n=96 | *P*-value | Included,  n=224 | Excluded,  n=140 | *P*-value |
| Male, % | 50 | 57 | 0.29 | 51 | 56 | 0.46 | 53 | 48 | 0.41 | 55 | 46 | 0.10 |
| Age, years | 25 (20, 32) | 25 (20, 31) | 0.69 | 25 (20, 31) | 25 (20, 31) | 0.95 | 25 (20, 31) | 25 (20, 31) | 0.58 | 25 (20, 31) | 25 (20, 31) | 0.56 |
| Height, cm | 148  (144, 151) | 147  (143, 152) | 0.47 | 147  (144, 152) | 148  (143, 152) | 0.73 | 147  (144, 150) | 147  (144, 152) | 0.24 | 147  (144, 151) | 148  (144, 152) | 0.18 |
| BMI, kg/m^2^ | 21 (20, 23) | 21 (20, 24) | 0.54 | 21 (20, 23) | 22 (20, 24) | 0.37 | 21 (19, 23) | 21 (20, 24) | 0.07 | 21 (20, 24) | 21 (19, 23) | 0.006 |
| Parity, n | 3 (2, 5) | 3 (2, 5) | 0.97 | 3 (2, 5) | 3 (2, 5) | 0.50 | 3 (2, 5) | 3 (2, 5) | 0.98 | 3 (2, 5) | 3 (2, 5) |  |
| Randomly assigned to praziquantel, % | 49 | 48 | 0.91 | 51 | 49 | 0.22 | 49 | 51 | 0.99 | 52 | 48 | 0.16 |
| *S. japonicum*, eggs/g | 10 (3, 33) | 10 (3, 23) | 0.46 | 10 (3, 33) | 8 (3, 23) | 0.30 | 10 (3, 30) | 10 (3, 30) | 0.97 | 13 (3, 33) | 10 (3, 27) | 0.06 |
| Hookworm, egg/g | 0 (0, 20) | 0 (0, 47) | 0.10 | 0 (0, 23) | 0 (0, 23) | 0.91 | 0 (0, 13) | 0 (0, 27) | 0.36 | 0 (0, 35) | 0 (0, 7) | 0.043 |

*P*-value for categorical variables obtained from Fisher’s exact tests. *P*-values for continuous variables obtained from Wilcoxon rank sum tests. A randomly selected subset was selected at each timepoint for comprehensive biomarker testing as resources allowed. There were no specific criteria for inclusion or exclusion.
